# Positive Surgical Margins in Clear Cell Renal Cell Carcinoma: Prognostic Impact and Implications for Risk Stratification and Adjuvant Therapy

**DOI:** 10.3390/jcm14113908

**Published:** 2025-06-02

**Authors:** Giuseppe Garofano, Cesare Saitta, Giacomo Musso, Margaret F. Meagher, Umberto Capitanio, Mai Dabbas, Natalie Birouty, Sanjana Karamcheti, Breanna Kim, Kit L. Yuen, Alessandro Larcher, Benjamin Baker, Riccardo Autorino, Savio D. Pandolfo, Francesco Montorsi, Alberto Saita, Massimo Lazzeri, Giovanni Lughezzani, Paolo Casale, Nicolò M. Buffi, Ithaar H. Derweesh

**Affiliations:** 1 Department of Urology, UC San Diego Health System, San Diego, CA 92121, USA; 2 Department of Biomedical Sciences, Humanitas University, Via Rita Levi Montalcini 4, 20072 Pieve Emanuele, MI, Italy; 3 IRCCS San Raffaele Scientific Institute, Urological Research Institute (URI), 20132 Milan, MI, Italy; 4 Department of Urology, University Vita-Salute San Raffaele, 20132 Milan, MI, Italy; 5 Department of Urology, Rush University Medical Center, Chicago, IL 60612, USA; 6 Department of Urology, University of L’Aquila, 67100 L’Aquila, AQ, Italy; 7 IRCCS Humanitas Research Hospital, Via Manzoni 56, 20089 Rozzano, MI, Italy

**Keywords:** adjuvant therapy, cancer staging, carcinoma, renal cell, nephrectomy, positive surgical margin, survival analysis

## Abstract

**Objectives:** To evaluate the prognostic impact of positive surgical margins (PSMs) after partial or radical nephrectomy for clear cell renal cell carcinoma (ccRCC) across AJCC stages and assess its relevance to adjuvant therapy eligibility, given that landmark trials excluded patients with PSMs. **Methods**: We conducted a retrospective study using the National Cancer Database, including 171,151 ccRCC patients treated with partial or radical nephrectomy (2004–2020). Patients receiving systemic therapy or with missing key data were excluded. OS was analyzed using Kaplan–Meier curves, log-rank tests, and multivariable Cox regression. Subgroup analyses compared T2 G2/G3 PSM vs. T2 G4 negative surgical margin (NSM) (N0/Nx M0) and assessed PSM impact within KEYNOTE-564 risk groups. **Results**: PSMs were present in 5.9% of patients and independently predicted worse OS (HR 1.43; *p* < 0.001). No OS difference was observed in AJCC stage I (*p* = 0.54), while stages II (*p* = 0.001), III, and IV (*p* < 0.001) showed poorer survival with PSMs. OS in patients with T2 G2/G3 tumors and PSMs was comparable to those with T2 G4 and NSMs (*p* = 0.69). Within the KEYNOTE-564 risk population, PSMs were associated with a 62% increased risk of death (HR 1.62; *p* < 0.001). **Conclusions**: PSMs are independently associated with worse OS in ccRCC. Their prognostic impact varies across AJCC stages, supporting the use of margin status to refine risk models, guide surveillance, and consider PSM patients for adjuvant trials.

## 1. Introduction

In recent years, adjuvant therapy with immune checkpoint inhibitors has emerged as a promising approach to the risk of recurrence in patients with clear cell renal cell carcinoma (ccRCC) at high risk [1,2,3,4,5]. The adoption of adjuvant immunotherapy in clinical guidelines [6] was driven by the KEYNOTE-564 trial, which showed improved disease-free and overall survival (OS) with pembrolizumab versus a placebo [7,8]. However, patients with positive surgical margins (PSMs) were systematically excluded from adjuvant trials, limiting evidence in this subgroup. Evidence from partial nephrectomy (PN) cohorts indicates that the presence of PSMs may be linked to increased risk of tumor recurrence and less favorable oncologic outcomes [9,10]. Although the literature on radical nephrectomy (RN) is more limited, recent findings suggest a similar pattern [11,12]. Moreover, PSMs are recognized as a marker of very high risk in American Urological Association (AUA) follow-up guidelines, underscoring their clinical relevance [13]. This discrepancy between risk stratification and trial design highlights a critical unmet need. To address this gap, we assessed the impact of PSMs on OS across AJCC pathological stages and subsequently focused on a high-risk population to evaluate their relevance to KEYNOTE-564-based selection.

## 2. Materials and Methods

### 2.1. Study Population and Patient Selection

**Figure 1:**
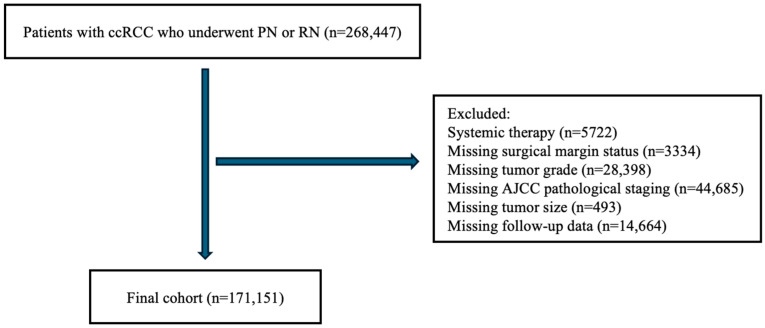
Selection criteria for the cohort. ccRCC: clear cell renal cell carcinoma; PN: partial nephrectomy; RN: radical nephrectomy; AJCC: American Joint Committee on Cancer.

This retrospective cohort study utilized the National Cancer Database (NCDB), a comprehensive, hospital-based registry that includes around 70% of all new cancer diagnoses in the US annually [14]. The inclusion criteria and selection flowchart are illustrated in Figure 1. We identified cases of RCC diagnosed between 2004 and 2020 using the International Classification of Diseases for Oncology, Third Edition (ICD-O-3) code C64.9, including patients who underwent PN or RN with clear cell histology (n = 268,447). We excluded patients who received systemic therapy to focus on a surgically managed population (n = 5722). Patients lacking information on surgical margin status (n = 3334), tumor grade (n = 28,398), AJCC pathological staging (n = 44,685), or tumor size (n = 493) were excluded from the final analysis. Finally, we excluded patients with missing follow-up data (n = 14,664). After applying these criteria, our final cohort consisted of 171,151 subjects.

### 2.2. Data Collected

The variables analyzed included demographic and clinical characteristics. Age at diagnosis and tumor size (measured in millimeters) were analyzed as continuous variables. Comorbidity burden was evaluated using the Charlson–Deyo Comorbidity Index (CCI), which was categorized as 0 for no comorbidities, 1 for a score of 1, 2 for a score of 2, and 3 for a score of 3 or more. Tumor-related characteristics included surgical margin status (categorized as positive or negative), tumor grade (G1 to G4), pathological T stage (pT1 to pT4), N stage (pN0/pNx vs. pN1), and M stage (M0 vs. M1). Staging was based on the 8th edition of the AJCC classification and categorized into four categories (I–IV). Additional variables included race (White, Black, Asian/Pacific Islander, Native American, and Other/Unknown), and surgical procedure (PN vs. RN).

### 2.3. Data Analysis

The primary objective of this study was to assess the prognostic impact of PSMs on OS in patients with ccRCC stratified by AJCC stage. Secondarily, we compared oncologic outcomes between patients with T2 G2/G3 tumors and PSMs, who are not eligible for adjuvant trials, and patients with T2 G4 tumors and negative surgical margins (NSMs), consistent with KEYNOTE-564 criteria (all N0/Nx M0). We then explored the prognostic impact of PSMs within the KEYNOTE-564-eligible population, stratifying by risk category. Descriptive statistics were reported as medians and interquartile ranges (IQRs) for continuous variables, and frequencies and percentages for categorical variables. Multivariable Cox proportional hazards models were used to estimate hazard ratios (HRs) and 95% confidence intervals (CIs) for all-cause mortality (ACM). Survival was analyzed using Kaplan–Meier curves and log-rank tests. We defined risk categories according to the KEYNOTE-564 criteria: intermediate-to-high risk (T2 with grade 4 or T3, with N0/Nx and M0) and high risk (T4 N0/Nx M0 or any T with N1 M0). Patients with metastatic disease were not included due to the lack of detailed information on metastasectomy. All statistical analyses were performed using Stata 18/SE (StataCorp, College Station, TX, USA). A two-sided *p*-value < 0.05 was considered statistically significant.

## 3. Results

### 3.1. Baseline Characteristics

Table 1 summarizes the baseline characteristics. Among 171,151 patients (median age 61; 61.46% male), 10,127 (5.9%) had PSMs. After a median follow-up of 70.2 months, 41,824 deaths were recorded. Compared to NSM patients, those with PSMs were older (63 vs. 61 years, *p* < 0.001), had larger tumors (50 mm vs. 42 mm, *p* < 0.001), more often had stage III–IV disease (54.12% vs. 22.59%, *p* < 0.001), and had high-grade tumors (G3–G4: 51.49% vs. 34.12%, *p* < 0.001). Partial nephrectomy was more common in the PSM group (50.08% vs. 41.88%, *p* < 0.001).

### 3.2. Overall Prognostic Impact of PSM

Table 2 presents the multivariable Cox regression for ACM. PSMs were associated with worse OS (HR 1.43; 95% CI: 1.38–1.49; *p* < 0.001). Age (per year: HR 1.046; 95% CI: 1.045–1.047; *p* < 0.001), tumor size (per mm: HR 1.001; 95% CI: 1.0009–1.0011; *p* < 0.001), and radical vs. partial nephrectomy (HR 1.47; 95% CI: 1.43–1.51; *p* < 0.001) were also significant. Compared to CCI 0, the HRs were 1.30 (95% CI: 1.28–1.34), 1.78 (95% CI: 1.72–1.84), and 2.27 (95% CI: 2.16–2.37) for CCI 1, 2, and ≥3, respectively (*p* < 0.001). AJCC stages II, III, and IV had HRs of 1.25 (95% CI: 1.21–1.30), 1.66 (95% CI: 1.61–1.70), and 4.74 (95% CI: 4.57–4.92) vs. stage I (*p* < 0.001). Grade 2 vs. 1 was not significant (*p* = 0.41), while grade 3 (HR 1.31; 95% CI: 1.26–1.36) and grade 4 (HR 2.04; 95% CI: 1.94–2.14) were associated with increased mortality (*p* < 0.001).

### 3.3. Kaplan–Meier Survival Analysis by AJCC Stage

**Figure 2:**
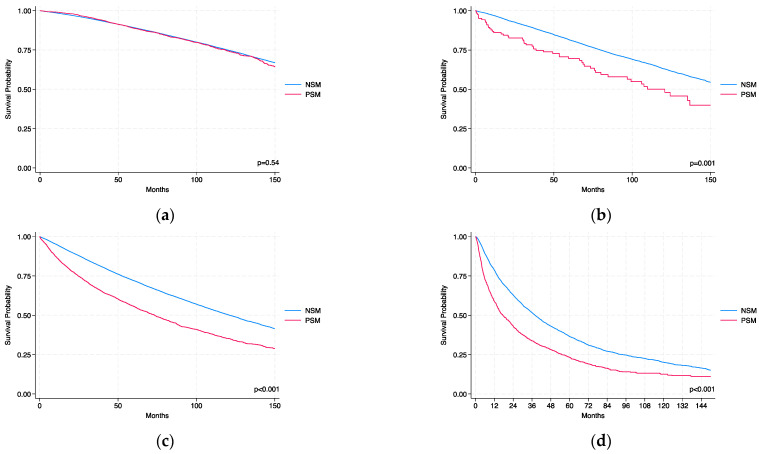
Kaplan–Meier curves for OS in AJCC stages I (**a**), II (**b**), III (**c**), and IV (**d**), stratified by surgical margin status. NSM: negative surgical margin; PSM: positive surgical margin.

Figure 2a–d illustrate Kaplan–Meier survival curves stratified by AJCC stage, showing the prognostic impact of PSMs on OS. In stage I, OS was comparable between PSMs and NSMs (log-rank *p* = 0.54). In stage II, patients with PSM had significantly worse OS compared to those with NSMs (log-rank *p* = 0.001), a difference that remained significant in stage III and IV (*p* < 0.001).

### 3.4. Comparison of T2 G2/G3 PSM vs. T2 G4 NSM

**Figure 3:**
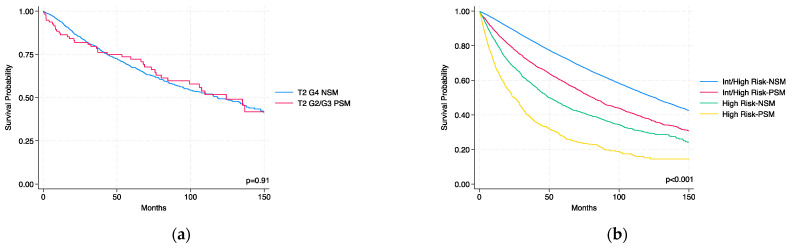
Kaplan–Meier curves for OS. (**a**) Survival comparison between patients with T2 G2/G3 tumors and positive surgical margins (PSMs) versus T2 G4 tumors with negative surgical margins (NSMs), all with N0/Nx M0 status. (**b**) OS stratified by KEYNOTE-564-defined risk categories (intermediate to high vs. high) and surgical margin status.

Figure 3a illustrates Kaplan–Meier survival curves comparing patients with T2 G2/G3 tumors and PSMs (n = 95) to those with T2 G4 tumors and NSMs (n = 1165), all with N0/Nx and M0 disease. The latter group represents one of the populations eligible for adjuvant therapy according to KEYNOTE-564 criteria. Kaplan–Meier analysis revealed no significant difference in OS between the two groups (log-rank *p* = 0.91). Table 2 reports the results of multivariable Cox regression, adjusted for age, comorbidity, surgical type, and tumor size, which confirmed the absence of a significant association (*p* = 0.69). These findings suggest that patients with intermediate-grade T2 tumors and PSMs may share a comparable oncologic risk with high-grade NSM patients currently considered for adjuvant therapy.

### 3.5. Survival Analysis Within KEYNOTE-564 Risk Categories

Figure 3b shows Kaplan–Meier survival curves stratified by KEYNOTE-564 risk category (n = 36,493) and surgical margin status. At 10 years, overall survival was 51.6% (95% CI: 50.8–52.4%) for intermediate-to-high-risk patients with NSMs, 37.9% (95% CI: 35.8–40.1%) for intermediate-to-high-risk patients with PSMs, 29.9% (95% CI: 27.4–32.4%) for high-risk patients with NSMs, and 15.2% (95% CI: 11.7–19.3%) for high-risk patients with PSMs. Table 2 reports results of the multivariable Cox regression analysis conducted within the KEYNOTE-564 cohort. Patients classified as high risk had more than double the risk of death compared to those in the intermediate-to-high-risk group (HR 2.40; 95% CI: 2.28–2.53; *p* < 0.001). Similarly, the presence of PSMs was associated with a 62% increased risk of death (HR 1.62; 95% CI: 1.55–1.70; *p* < 0.001). Among this high-risk population, age (HR 1.034 per year; 95% CI: 1.032–1.036), comorbidity (CCI ≥3: HR 1.75, 95% CI: 1.62–1.88; *p* < 0.001), tumor size (HR 1.001 per mm; 95% CI: 1.0009–1.0011), and type of surgery (radical vs. partial nephrectomy: HR 1.61; 95% CI: 1.50–1.72) were also independently associated with ACM.

## 4. Discussion

This study provides the largest contemporary analysis of the prognostic significance of PSMs in ccRCC, including over 170,000 surgically treated patients. In contrast to prior studies limited to specific surgical approaches or early-stage tumors, this analysis examines the prognostic impact of PSMs following both partial and radical nephrectomy, including all AJCC pathological stages. This broader inclusion enables a more comprehensive assessment of real-world heterogeneity among surgically treated patients, particularly those eligible for adjuvant therapy [15]. While current adjuvant therapy trials systematically exclude patients with PSMs, our findings highlight that PSMs are independently associated with worse survival and may identify a subgroup of patients with oncologic risk comparable to that of trial-eligible populations. Among high-risk individuals, PSMs further increase mortality, suggesting additive prognostic value. These findings support margin status as a relevant factor for patient selection in future strategies.

The status of surgical margins is considered an important indicator of surgical quality, and its assessment has become an integral part of postoperative outcome evaluation in RCC surgery. In this context, structured frameworks such as the Trifecta [16] and the Margin, Ischemia, and Complications (MIC) score [17] have emerged as valuable tools for defining surgical success. These models aim to capture the multifactorial nature of surgical performance by considering multiple relevant factors, with surgical margin status being a central component in both. Consistently with this perspective, the AUA guidelines emphasize that the primary goal of PN is to achieve NSMs. The guidelines state that patients with microscopic PSMs should be considered at higher risk and managed with increased clinical vigilance, including more intensive follow-up, while macroscopic PSMs are classified as very high risk due to the evident presence of residual disease and the significantly higher likelihood of local recurrence [13].

The prognostic significance of PSMs has been studied predominantly in the context of PN. In a meta-analysis published by Ficarra et al. [18] and including over 45,000 patients, PSMs were found to be significantly associated with increased risk of recurrence. However, this study reported no significant association between PSMs and cancer-specific survival (CSS) or OS, suggesting a limited impact on long-term mortality in the general PN population. Emerging evidence, however, suggests a potentially greater prognostic value of PSMs. More recently, Hakam et al. [19] conducted a larger meta-analysis including over 100,000 patients who underwent nephron-sparing surgery. They found that PSMs were significantly associated with increased risk of local recurrence (HR 6.11, *p* < 0.001), metastasis (HR 3.29, *p* = 0.01), and ACM (HR 1.30, *p* < 0.01), despite variable certainty of evidence across outcomes. Although both meta-analyses are limited to PN, recent studies have also begun to clarify the impact of PSMs following RN. In a retrospective cohort study of 714 patients, Abu-Ghanem et al. [11] found that PSMs were independently associated with increased risk of local recurrence (HR 4.8, *p* = 0.01) and cancer-specific mortality (HR 2.4, *p* = 0.03), although no significant association with OS was observed (HR 1.3, *p* = 0.5). Similarly, in a single-institution analysis of 77 patients with T3 RCC treated with RN, Morris et al. [12] found that PSMs were significantly associated with inferior RFS at both 3 years and 5 years (*p* = 0.01). Although OS appeared lower in the PSM group (5-year OS: 85.7% vs. 93.8%), the difference did not reach statistical significance (*p* = 0.34).

Based on our analysis of more than 170,000 surgically treated ccRCC patients, PSMs were independently associated with an increased risk of death (HR 1.43; *p* < 0.001), even after adjusting for a comprehensive set of clinical and pathological covariates. Moreover, in a focused subgroup analysis, patients with T2 grade 2 or 3 tumors and PSMs showed survival outcomes similar to those of T2 grade 4 patients with NSMs, a group currently considered eligible for adjuvant immunotherapy according to KEYNOTE-564 criteria (*p* = 0.69). These findings underscore the limitations of relying exclusively on TNM stage and tumor grade to stratify patients for postoperative treatment and suggest that incorporating additional factors such as surgical margin status may provide a more effective and inclusive strategy to identify high-risk patients who could benefit from adjuvant therapy.

The prognostic significance of PSMs is increasingly recognized as being influenced by tumor stage. Shah et al. [20] conducted a multi-institutional study evaluating RFS after PN in 1240 patients with localized RCC, stratified by risk group. Their findings demonstrated that PSMs significantly increased the risk of recurrence in high-risk patients (pT2-3a and/or grade III–IV; HR = 7.48, *p* < 0.001) but had no significant impact in low-risk patients (pT1 and grade I–II; *p* = 0.647). Similarly, Kang et al. [21] analyzed 1813 patients with pT1 clear cell RCC treated with PN and found that PSMs did not influence RFS (*p* = 0.566), with no local recurrences observed among patients with PSMs. Supporting these observations, our Kaplan–Meier analysis by AJCC stage (Figure 2a–d) showed no survival difference between PSMs and NSMs in stage I (log-rank *p* = 0.54). By contrast, PSMs were associated with significantly worse survival in stage II (*p* = 0.001), with an even more pronounced difference observed in stages III and IV (*p* < 0.001). In this context, our analysis stratified by KEYNOTE-564 risk categories further refines the prognostic interpretation of PSMs. The combination of high-risk features and PSMs identifies a subgroup with reduced long-term survival (HR 1.62; 95% CI: 1.55–1.70), underscoring the cumulative effect of biological aggressiveness and incomplete resection. These results suggest that PSMs have limited prognostic relevance in low-risk tumors yet become clinically meaningful in more aggressive tumors. This stage-dependent effect supports a risk-adapted approach to postoperative surveillance and management, potentially allowing for less intensive follow-up in low-risk patients with PSMs and emphasizing the need for close monitoring in high-risk cases.

We restricted analysis to ccRCC to align with clinical trial populations and ensure histologic homogeneity. Notably, the KEYNOTE-564 trial enrolled only patients with clear cell histology. Furthermore, a recent multi-institutional study by Hulin et al. [22], conducted on a cohort of 1115 patients who underwent partial nephrectomy, investigated the prognostic significance of PSMs across different histological subtypes. In their histological subgroup analysis, patients with ccRCC and PSMs exhibited a significantly shorter DFS compared to those with NSMs (*p* = 0.03), while the difference in DFS was not statistically significant for papillary RCC (*p* = 0.09) or chromophobe RCC (*p* = 0.98). These findings reinforce the biologically distinct behavior of ccRCC and support the use of a histology-restricted design to avoid confounding and enhance clinical relevance.

Our study presents several limitations. The NCDB is hospital-based, which may restrict the generalizability of our findings to the broader population of patients with RCC. Its retrospective nature may introduce selection bias, particularly due to the exclusion of patients with missing values for key clinical and pathological variables and lack of data on surgical technique and surgeon experience. Predictors of surgical difficulty, such as PADUA or RENAL nephrometry scores [23,24], are not available in the NCDB, potentially limiting adjustment for tumor complexity. No central pathology review may lead to variability in PSM classification. Moreover, the database does not distinguish between microscopic and macroscopic PSMs. Information on whether patients received further treatment after PSMs, such as reoperation or delayed therapy, is also unavailable. The NCDB reports only OS and lacks data on cause-specific mortality or recurrence, limiting evaluation of cancer-specific outcomes. As such, further investigation and validation with datasets which include data on recurrence and cancer-specific mortality are required. Despite these limitations, the large sample size of the NCDB allows for a robust analysis of the prognostic implications of PSMs across pathological stages and risk groups, offering valuable insights into a population often excluded from prospective clinical trials.

## 5. Conclusions

PSMs are independently associated with worse OS in patients with ccRCC. However, their prognostic impact is influenced by additional factors such as AJCC stage. Incorporating margin status with established risk factors may help better identify patients at greatest risk and call for an update of guidelines which recommend intensified surveillance. Our findings also call for prospective studies to evaluate salvage or adjuvant therapy in patients with PSMs at greatest risk for progression.

## Figures and Tables

**Table 1:** Demographics and clinical characteristics.

Variable	Overall (n = 171,151)	PSM (n = 10,127)	NSM (n = 161,024)	*p*-Value	Test
**Age**, years: median (IQR)	61 (52–69)	63 (55–71)	61 (52–69)	<0.001	#
**Gender, n (%)**				<0.001	*
Male	105,196 (61.46)	6518 (64.36)	98,678 (61.28)		
Female	65,955 (38.54)	3609 (35.64)	62,346 (38.72)		
**Race, n (%)**				<0.001	*
White	150,827 (88.13)	9037 (89.24)	141,790 (88.06)		
Black	11,585 (6.77)	588 (5.81)	10,997 (6.83)		
Native American	1170 (0.68)	82 (0.81)	1088 (0.68)		
Asian/Pacific Islander	4001 (2.34)	224 (2.21)	3777 (2.35)		
Other/Unknown	3568 (2.08)	196 (1.94)	3372 (2.09)		
**CCI**				<0.001	*
0	114,708 (67.02)	6561 (64.79)	108,147 (67.16)		
1	37,185 (21.73)	2309 (22.80)	34,876 (21.66)		
2	11,763 (6.87)	752 (7.43)	11,011 (6.84)		
≥3	7495 (4.38)	505 (4.99)	6990 (4.34)		
**Tumor size**, mm: median (IQR)	42 (27–65)	50 (29–91)	42 (27–65)	<0.001	#
**Surgery type**				<0.001	*
Partial nephrectomy	72,507 (42.36)	5072 (50.08)	67,435 (41.88)		
Radical nephrectomy	98,644 (57.64)	5055 (49.92)	93,589 (58.12)		
**Pathological T stage, n (%)**				<0.001	*
T1	116,317 (67.96)	4582 (45.25)	111,735 (69.39)		
T2	15,267 (8.92)	168 (1.66)	15,099 (9.38)		
T3	38,411 (22.44)	4905 (48.43)	33,506 (20.81)		
T4	1156 (0.68)	472 (4.66)	684 (0.42)		
**Pathological N stage, n (%)**				<0.001	*
N0/Nx	167,903 (98.10)	9261 (91.45)	158,642 (98.52)		
N1	3248 (1.90)	866 (8.55)	2382 (1.48)		
**Metastatic status, n (%)**				<0.001	*
M0	164,594 (96.17)	8943 (88.31)	155,651 (96.66)		
M1	6557 (3.83)	1184 (11.69)	5373 (3.34)		
**AJCC stage, n (%)**				<0.001	*
I	115,167 (67.29)	4523 (44.66)	110,644 (68.71)		
II	14,119 (8.25)	122 (1.20)	13,997 (8.69)		
III	34,575 (20.20)	4026 (39.76)	30,549 (18.97)		
IV	7290 (4.26)	1456 (14.38)	5834 (3.62)		
**Grade, n (%)**				<0.001	*
G1	19,588 (11.44)	782 (7.72)	18,806 (11.68)		
G2	91,399 (53.40)	4130 (40.78)	87,269 (54.20)		
G3	48,236 (28.18)	3418 (33.75)	44,818 (27.83)		
G4	11,928 (6.97)	1797 (17.74)	10,131 (6.29)		

# Wilcoxon rank-sum test; * Pearson’s chi-squared test; PSM: positive surgical margin; NSM: negative surgical margin; IQR: interquartile range; CCI: Charlson–Deyo Comorbidity Index; AJCC: American Joint Committee on Cancer.

**Table 2:** Multivariable Cox regression for all-cause mortality: (a) overall cohort; (b) T2 G2/G3 with PSM vs. T2 G4 with NSM (N0/Nx M0); (c) KEYNOTE-564 cohort.

(**a**)			
**Variable**	**HR**	***p*** **-Value**	**95% CI**
**Surgical margin**			
NSM	Reference		
PSM	1.43	<0.001	1.38–1.49
**Age**	1.046	<0.001	1.045–1.047
**CCI**			
0	Reference		
1	1.30	<0.001	1.28–1.34
2	1.78	<0.001	1.72–1.84
≥3	2.27	<0.001	2.16–2.37
**Tumor size (mm)**	1.001	<0.001	1.0009–1.0011
**Surgery type**			
Partial nephrectomy	Reference		
Radical nephrectomy	1.47	<0.001	1.43–1.51
**AJCC stage**			
I	Reference		
II	1.25	<0.001	1.21–1.30
III	1.66	<0.001	1.61–1.70
IV	4.74	<0.001	4.57–4.92
**Grade**			
G1	Reference		
G2	1.02	0.41	0.98–1.06
G3	1.31	<0.001	1.26–1.36
G4	2.04	<0.001	1.94–2.14
(**b**)			
**Variable**	**HR**	***p*** **-Value**	**95% CI**
**T2 subgroups**			
T2 G4 NSM	Reference		
T2 G2/G3 PSM	1.08	0.69	0.74–1.57
**Age**	1.03	<0.001	1.03–1.04
**CCI**			
0	Reference		
1	1.06	0.63	0.84–1.32
2	1.04	0.83	0.70–1.56
≥3	2.15	<0.001	1.42–3.23
**Tumor size (mm)**	0.99	0.90	0.98–1.00
**Surgery type**			
Partial nephrectomy	Reference		
Radical nephrectomy	1.35	0.11	0.94–1.95
(**c**)			
**Variable**			
**Keynote group**			
Intermediate-to-High Risk	Reference		
High Risk	2.40	<0.001	2.28–2.53
**Surgical margin**			
NSM	Reference		
PSM	1.62	<0.001	1.55–1.70
**Age**	1.034	<0.001	1.032–1.036
**CCI**			
0	Reference		
1	1.25	<0.001	1.20–1.30
2	1.43	<0.001	1.34–1.52
≥3	1.75	<0.001	1.62–1.88
**Tumor size (mm)**	1.001	<0.001	1.0009–1.0011
**Surgery type**			
Partial nephrectomy	Reference		
Radical nephrectomy	1.61	<0.001	1.50–1.72

HR: hazard ratio; CI: confidence interval; NSM: negative surgical margin; PSM: positive surgical margin; CCI: Charlson–Deyo Comorbidity Index; AJCC: American Joint Committee on Cancer.

## Data Availability

Access to the National Cancer Database is restricted and granted only to approved institutions and researchers. Due to these restrictions, the dataset cannot be publicly shared.
